# Discrete Element Modeling of Instability Mechanisms of Unbound Permeable Aggregate Base Materials in Triaxial Compression

**DOI:** 10.3390/ma15082716

**Published:** 2022-04-07

**Authors:** Yuanjie Xiao, Xiaoming Wang, Qunding Yu, Juanjuan Ren, Wenjun Hua, Ralina Mustafina, Fuguang Zhang, Huaiping Feng, Tongwen Zhang

**Affiliations:** 1 School of Civil Engineering, Central South University, Changsha 410075, China; yjxiao@csu.edu.cn (Y.X.); xiaoming-wang@csu.edu.cn (X.W.); qdyucc@126.com (Q.Y.); wenjun.hua@csu.edu.cn (W.H.); ralina.free@mail.ru (R.M.); 2 Ministry of Education Key Laboratory of Engineering Structures of Heavy Haul Railway, Central South University, Changsha 410075, China; 3 Urban Rail and Underground Engineering Design and Research Institute, China Railway Fourth Survey and Design Institute Group Co., Ltd., Wuhan 430063, China; 4 School of Civil Engineering, Southwest Jiaotong University, Chengdu 610031, China; 5 State Key Laboratory of Mechanical Behavior and System Safety of Traffic Engineering Structures, Shijiazhuang Tiedao University, Shijiazhuang 050043, China; fenghuaiping@stdu.edu.cn; 6 School of Railway Engineering, Hunan Technical College of Railway High-Speed, Hengyang 421002, China

**Keywords:** unbound permeable aggregate base, gradation, discrete element method, shear strength behavior, fabric evolution, particle movement

## Abstract

Unbound permeable aggregate base (UPAB) materials with strong load-transmitting skeleton yet adequate inter-connected pores are desired for use in the sponge-city initiative. However, the micro-scale fabric evolution and instability mechanism of macroscopic strength behavior of such UPAB materials still remain unclear. In this study, virtual monotonic triaxial compression tests were conducted by using the discrete element method (DEM) modeling approach on specimens with different gradations quantified by the parameter of gravel-to-sand ratio (G/S). The realistic aggregate particle shape and inter-particle contact behavior were properly considered in the DEM model. The micromechanical mechanisms of the shearing failure of such UPAB materials and their evolution characteristics with G/S values were disclosed from contact force chains, microstructures, and particle motion. It was found that the proportion of rotating particles in the specimens decreased and the proportion of relative sliding between particles increased as the content of fine particles decreased. The plastic yielding of the specimens originated from the failure of contact force chains and the occurrence of the relative motion between particles, while the final instability was manifested by the large-scale relative motion among particles along the failure plane (i.e., changes in the internal particle topology). By comparing the macroscopic strength, microstructure evolution, and particle motion characteristics of the specimens with different G/S values, it was found that the specimens with G/S value of 1.8 performed the best, and that the G/S value of 1.8 could be regarded as the threshold for separating floating dense and skeletal gap type packing structures. The variation of Euler angles of rotating particles was significantly reduced in the particle size range of 4.75 mm to 9.50 mm, indicating that this size range separates most of the particles from rolling and sliding. Since particle rolling and sliding behavior are directly related to shear strength, this validates the rationality of the parameter G/S for controlling and optimizing gradations from the perspective of particle movement. The findings could provide theoretical basis and technical guidance for the effective design and efficient utilization of UPAB materials.

## 1. Introduction

With the frequent occurrence of extreme rainfall events in the world, flood and waterlogging disasters have become more prominent, especially in urban areas. The initiative and construction of sponge cities are among the technical measures to mitigate such disasters. Unbound permeable aggregate base (UPAB) is widely used as drainage layers due to its advantages of relatively larger air voids and, thus, better permeability (or drainability) as compared to conventional dense-graded aggregate bases [1]. Kibert (1994) laid down the foundation for sustainable construction (SC) practice and established SC around resource minimization and reuse, the use of renewable and recyclable resources, and minimizing carbon footprint. The efficient use of UPAB materials, in lieu of cement- or asphalt-stabilized materials, in constructing pavement drainage layers echoes with the currently agreed common goals of SC: reducing carbon footprint, eliminating environmentally harmful materials, improving resource efficiency, and conserving resources (land and raw materials) [2]. However, the majority of existing studies focused on the design and construction of permeable surface layers, such as porous asphalt concrete [3], porous Portland cement concrete, and permeable wearing courses (e.g., open-graded friction courses (OGFCs)) [4,5]. In addition to providing uniform and stable support for the surface layer, UPAB also serves the functionality of transmitting repeated traffic load to underlying layers, providing enough drainage channels, and achieving better cost-effectiveness. It has thus been increasingly used in permeable pavement foundation in recent years. However, how to cost-effectively balance mechanical properties (e.g., shear strength, resilient modulus, and permanent deformation) and hydraulic features (e.g., large voids, inter-connected pore structures, and high permeability) remains to be one of the key challenges [6]. For unbound aggregates, the shear strength properties are better correlated with permanent deformation and settlement than a resilient modulus [7,8]. Therefore, in order to avoid the instability and excessive deformation of UPAB layers, it is of great theoretical and practical significance to study their shear strength behavior and its micro-scale evolution law with microstructural fabrics and particle movement and then recommend optimal gradation design.

For the UPAB materials, a robust gradation design must ensure that the assembly of unbound aggregates has sufficient shear strength yet meanwhile the desired permeability. However, the content of fine particles in the UPAB materials has an important effect on the shear strength and permeability. Ling et al. (2017) [9] conducted laboratory triaxial tests on coarse-grained soils and found that the cohesion decreased and the angle of internal friction increased with increasing content of fine particles; in contrast, Bouziane et al. (2020) [10] showed from the results of a study on sandy soils that the cohesion increased and the angle of internal friction decreased with increasing content of fine particles. It seems that the influence of fine particles on the macroscopic shear strength behavior of different coarse-grained materials remains ambiguous, and that few studies have revealed the meso-scale and micromechanical mechanisms of shearing instability (or failure) affected by fine particles. The content of fine particles is reflected in the gradation design of UPAB materials; therefore, studying the microscale fabric evolution and instability mechanism of shear strength behavior of UPAB materials with different gradations is of great significance. Xiao et al. (2012) [11] found that the “Gravel to Sand ratio (G/S)” parameter proposed based on the particle packing theory can effectively optimize the fine fraction of both conventional virgin and recycled unbound granular materials (UGMs) and can be used to quantify the effect of fine fraction on shear strength behavior of such UGMs. Their findings have been increasingly substantiated in recent studies [12,13,14]. For instance, Wilde et al. (2016) [13] found that the optimal range of G/S values in unbound granular pavement base/subbase materials was 0.8–1.4; however, they did not examine the shear strength of those materials with different G/S values. Qamhia and Tutumluer (2021) also reported that the G/S value needs to be controlled and limited to 1.3–1.9 in order to ensure the stability of the daylighted subbase [14]. From the literature reviewed above, it becomes clear that the content of fine particles has an important influence on the shear strength behavior of coarse-grained soils, especially UPAB materials (where too much fine fraction causes a loss of permeability, while too little fine fraction may compromise shear strength). A proper proportion of large coarse (gravel size) aggregates in contact is needed to maximize shear strength, while a proper content of fine (sand size) aggregates filling the voids of large coarse aggregates is needed to reduce permanent deformation (or settlement). Therefore, it remains indispensable to study the effect of fine fraction on macroscopic shear strength behavior, microscale fabric evolution, and instability mechanism, and to further validate the role of G/S parameter in controlling the shear strength behavior of UPAB materials.

With the rapid development of numerical simulation technology, the discrete element method (DEM) has been widely used to provide micromechanical insights into mechanical behavior of geomaterials [15,16,17,18]. Unlike laboratory testing methods, the DEM has unique advantages in revealing microstructural characteristics and micromechanical mechanisms (e.g., Ng 2009; Xiao and Tutumluer, 2017). Marczwska et al. (2016) [15,19,20] investigated from the DEM simulations macroscopic mechanical responses and related meso-scale mechanisms of UGMs during compaction. Tang et al. (2017) [21] revealed from DEM simulations the micromechanical mechanism of macroscopic plasticity development in UGMs. Qin et al. (2021) [22] found from DEM study that the concentration phenomenon of meso-scale particle contact force chains is the main reason for the different damage patterns. It clearly shows that the unique advantage of the DEM is that it can probe meso-scale nature of macroscopic behavior of UGMs and reveal microscopic mechanisms attributable to their macroscopic mechanical responses [23,24,25]. However, there currently exists few DEM studies on UPAB materials, especially those on the effect of fine particles on shear strength behavior of UPAB materials from the perspectives of meso-scale mechanisms and particle packing based gradation optimization.

UPAB materials are increasingly used in pervious (or porous) pavement layers due to large air void structure and thus good permeability characteristics. However, there is still a lack of in-depth study on their shear strength characteristics and micromechanical mechanisms of shearing instability (or failure) under different gradations. The primary objective of this study was to disclose the meso-scale instability (or shear failure) mechanisms of UPAB materials with varying gradations (or particle packing structures) in triaxial compression conditions, as manifested by the evolution of fabric anisotropy, microstructure change, and particle movement. To achieve this goal, a series of laboratory monotonic triaxial compression tests were conducted on UPAB specimens with different gradations to investigate the effect of gradation on macroscopic shear strength characteristics. The accompanying 3D DEM simulations were subsequently performed with realistic irregular particle shape considered to disclose micromechanical mechanisms of shearing instability under different gradations. Finally, the angle of anisotropy, microstructural fabric, and particle movement of the particulate granular system of UPAB materials were comparatively analyzed from DEM simulation results. The findings are expected to promote the gradation optimization of UPAB materials and their potential use in constructing long-lasting, high-performance pavement drainage layers for sponge-city initiatives.

## 2. Materials and Methods

### 2.1. Materials

**Figure 1:**
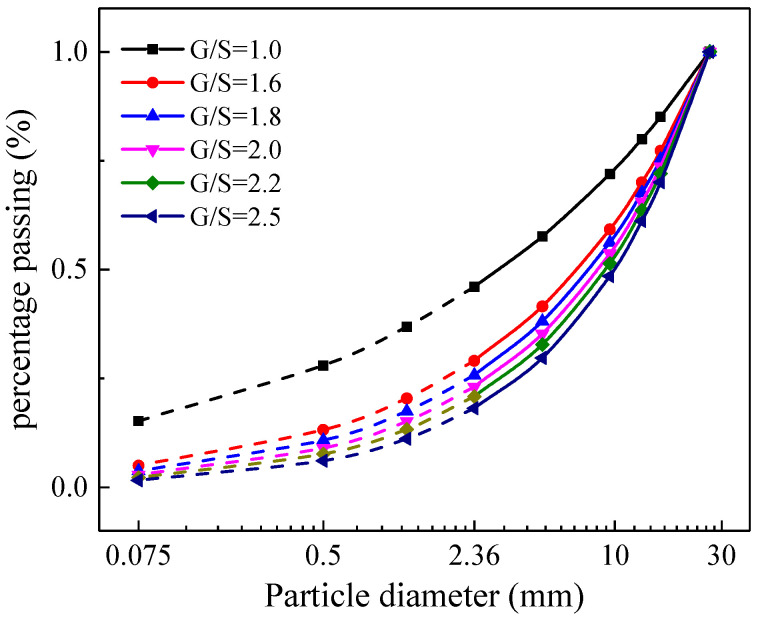
Gradation curves of UPAB materials studied.

The particle-size distribution (gradation) of raw aggregate materials was determined from sieve analysis by following the related Chinese standard (similar to ASTM D6913/D6913M-17). and shown in Figure 1. Specimens with such gradations were prepared and subjected to laboratory monotonic triaxial compression tests. In this study, the gravel-to-sand ratio (G/S) parameter was used for gradation design. The G/S parameter was first proposed by Sánchez (2007) based on the Talbot function for the gradation design of hot mix asphalt and then improved and extended to the gradation design of UPAB/subbase materials [6]. It was found that the G/S parameter can be used to control shear strength and other mechanical properties of UPAB/subbase materials reasonably well. The G/S parameter can be calculated according to Equation (1), where the shape parameter n of the gradation curve can be determined by the Talbot function expressed in Equation (2). Note that all the sieve sizes of the gradation curve and their percent passing values are used to calculate the G/S parameter. The G/S values of six different gradations designed are calculated as 1.0, 1.6, 1.8, 2.0, 2.2 and 2.5, respectively. Other relevant gradation parameters are listed in Table 1.

(1) $$\frac{G}{S}=\frac{1-P_{4.75}}{1-\left(1-P_{4.75}\right)-P_{0.075}}=\frac{D_{max}^{n}-4.75^{n}}{4.75^{n}-0.075^{n}}$$

(2) $$P_{i}={\left(\frac{d_{i}}{D_{max}}\right)}^{n}$$

where $P_{i}$ is the percentage of materials passing the i-th sieve, $d_{i}$ is the opening size of the i-th sieve (mm), $D_{max}$ is the maximum particle size (mm), and n is the shape parameter of the gradation curve.

### 2.2. Laboratory Testing Procedures

To determine the optimal moisture content (OMC) and maximum dry density (MDD) for each of the five different gradations, laboratory compaction tests were conducted by following the related Chinese standard (JTG 3430-2020) [22], of which the procedure is quite similar to that of the Modified Proctor compaction (AASHTO T180). Specifically, the compaction specimens of 152 mm in diameter and 120 mm in height were prepared in 3 lifts (or sublayers) with each lift subjected to 98 blows of a 4.5 kg hammer falling freely from a height of 450 mm, thus applying the impact energy per unit volume of 2691 kJ/cm^3^. The compaction curves of gravitational moisture content versus achieved dry density were skipped for brevity, while the OMC and MDD results obtained as such are summarized in Table 2. As compared to conventional dense-graded aggregate materials, the UPAB materials are relatively more difficult to compact due to larger air voids; therefore, the minimum degree of compaction was set as 90%, of which higher values were found to potentially cause considerable particle breakage.

**Figure 2:**
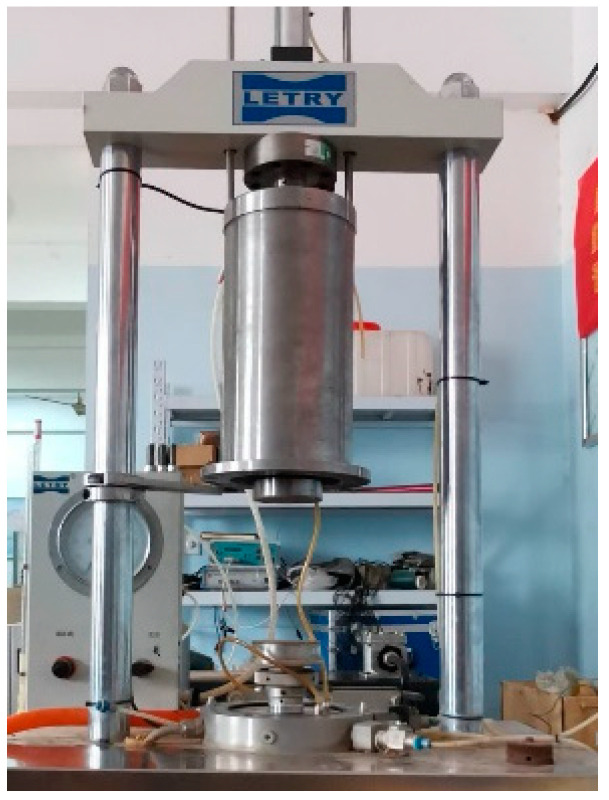
The STD-50 triaxial testing apparatus.

**Figure 3:**
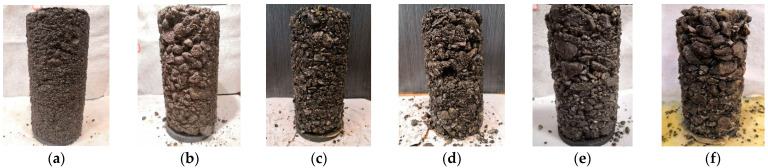
The close-up photos of laboratory specimens with varying G/S values: (**a**) G/S = 1.0; (**b**) G/S = 1.6; (**c**) G/S = 1.8; (**d**) G/S = 2.0; (**e**) G/S = 2.2; and (**f**) G/S = 2.5.

The shear strength of unbonded granular materials is an important mechanical property that controls the rate of permanent deformation of unbonded granular materials and affects the performance of the pavement [26,27,28]. Therefore, it is important to carry out monotonic triaxial compression tests on such UPAB materials to examine their shear strength behavior. The related Chinese standard (JTG 3430-2020) was followed to better simulate any possible failure condition of an in-service pavement layer under the dynamic application of a moving wheel load, which is quite similar to those described in previous studies [29,30,31,32,33]. The electro-hydraulic servo triaxial apparatus (Model No. STD-50) used in this study is shown in Figure 2. The triaxial specimens of six different gradations (see Figure 1) were prepared at their optimal conditions according to the obtained OMC and MDD results (see Table 2), respectively. The dimensions of the triaxial specimens were 100 mm in diameter and 200 mm in height, as illustrated in Figure 3. Three representative levels of confining pressure were preselected based on the typical stress states in pavement base/subbase layers [32], i.e., 50 kPa, 100 kPa and 200 kPa, while the peak deviator stress at failure was recorded under each confining pressure level. Note that the relatively higher confining pressure level selected for UPAB materials in this study was due to the equipment limitation and meant to represent thinly surfaced or unsurfaced pavement applications. The loading rate was set to 1 mm/min, and previous studies reported that the strength results from monotonic loading tests are not sensitive to the shearing rate adopted [31]. It is worth mentioning that the laboratory repeated load triaxial (RLT) tests were also conducted on such UPAB materials to characterize their dynamic behavior including resilient modulus and permanent deformation, but those results were out of the scope and skipped herein. Instead, only the shear strength results were presented for the purpose of calibrating micromechanical input parameters of the DEM model to be developed.

The saturated permeability values of UPAB materials tested were measured to all exceed 0.803 cm/s, which is greater than the minimum value of 0.347 cm/s specified by related Chinese standard. The porosity values also ranged from 0.2 to 0.3. Since the permeability part is out of the scope of this study, more details can be found elsewhere [33].

### 2.3. Particle Shape Acquisition and Quantification

**Figure 4:**
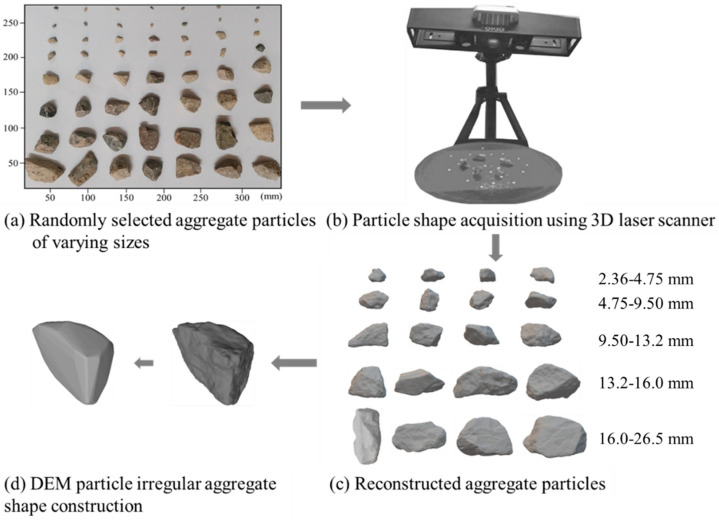
Illustration of the flowcharts of particle shape acquisition and quantification: (**a**) Randomly selected aggregate particles of varying sizes; (**b**) particle shape acquisition using 3D laser scanner; (**c**) Reconstructed aggregate particles; and (**d**) DEM particle irregular aggregate shape construction.

To date, many shape indices, either two- or three-dimensional, have been proposed to characterize and quantify irregular particle shape, including form, angularity, and surface texture. Zhang et al. (2020) [34] pointed out that shape factors such as elongation, flatness and sphericity have an important effect on the fragmentation of aggregates, i.e., the greater the elongation and flatness indices are, the more likely aggregate particles are to break during shearing and the shear strength is to reduce. Tutumluer (2008) [35] reported that convexity and ellipsoidal convexity indices contribute significantly to the strength and stability of aggregate assembly, while surface roughness tends to reduce the spreading effect of aggregate assembly by increasing the friction among individual aggregate particles; in other words, the greater such shape indices are, the higher the shear strength of the aggregate assembly is. Due to the important effects of particle shape on shear strength and other mechanical properties, aggregate particles with similar shape characteristics were intentionally selected in this study for use in the laboratory monotonic triaxial compression tests so that the influence of particle shape variation on the strength evolution characteristics and microscopic mechanisms can be minimized. To quantify shape characteristics of aggregate particles used in this study, representative particles ranging from 2.36 mm to 26.5 mm were randomly selected with their real 3D shape captured by the laser scanner and reconstructed for subsequent use in the library of particle shape of the DEM model, as shown in Figure 4.

**Figure 5:**
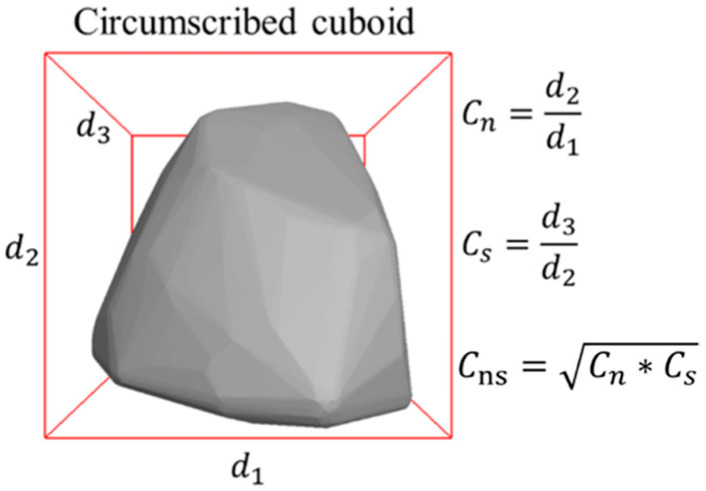
The diagram defining typical particle shape indices C_n_, C_s_, and C_ns_.

**Figure 6:**
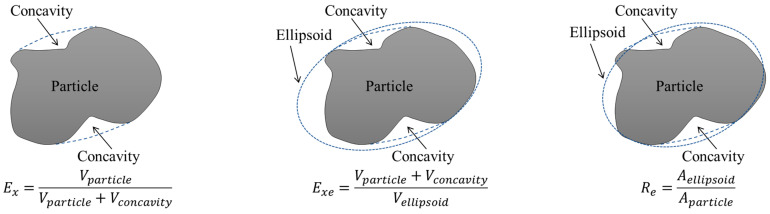
The diagrams illustrating the calculations of form, roundness, and surface roughness indices.

Typical particle shape indices formulated in Equations (3) to (7) were computed and used as the benchmark to realistically generate virtual particles in the DEM model. Specifically, the calculation formulas of morphological indices C_n_, C_s_, and C_ns_ and related illustrative diagrams are shown in Equations (3) to (4) and Figure 5, respectively [36]. The calculation formulas of Convexity $E_{x}$ and ellipsoidal convexity $E_{xe}$ are given in Equations (5) to (6) and related illustrative diagrams are shown in Figure 5 [37,38]. The surface area ratio of the ellipsoid is defined as the roughness index $R_{e}$ [39], of which the calculation formula is given in Equation (7) and the related illustrative diagram is shown in Figure 6. The closer the values of elongation $C_{n}$ and Flatness $C_{s}$ are to 0, the more obvious the needle and flake shape of the entire particle is. For deformity factor $C_{ns}$ values closer to 0, the greater the degree of deformity of particle shape. For convexity $E_{x}$ and ellipsoid convexity $E_{xe}$ values closer to 1, the angular characteristics of particles are more obvious. The calculation results of such typical shape indices are listed in Table 3. The results show that the selected aggregate particles have a wide range of morphological characteristics, mainly convex particles, and the angular characteristics are not obvious, but the surface has a certain degree of roughness; in addition, the selected particles do not have a certain extreme particle morphological characteristic, which effectively avoids the influence of a single particle morphological characteristics on the overall strength of the particles.

(3) $$C_{n}=\frac{d_{2}}{d_{1}},\;C_{s}=\frac{d_{3}}{d_{2}}$$

(4) $$C_{ns}=\sqrt{C_{n}\cdot C_{s}}$$

(5) $$E_{x}=\frac{V}{V_{x}}$$

(6) $$E_{xe}=\frac{V_{x}}{\sqrt{\frac{3}{4\pi }}A_{S}^{1.5}\cdot \frac{{\left(C_{n}\right)}^{0.5}\cdot C_{S}}{{\left(1+C_{S}+C_{n}C_{S}\right)}^{1.5}}}$$

(7) $$R_{e}=\frac{{\left(\frac{4\pi }{3}\right)}^{\frac{1}{3}}\cdot V^{\frac{2}{3}}\cdot \left(1+C_{S}+C_{n}C_{S}\right)}{A_{S}\cdot {\left(C_{n}\right)}^{\frac{1}{3}}\cdot {\left(C_{S}\right)}^{\frac{2}{3}}}$$

where $d_{1}$, $d_{2}$, and $d_{3}$ denote the long axis, intermediate axis, and short axis of each particle, respectively, in mm; $A_{S}$ denotes the surface area of each particle in mm^2^; V denotes the volume of each particle in mm^3^; $R_{e}$ denotes the radius of the maximum inscribed sphere of each particle in mm; and $V_{x}$ denotes the convex hull volume of each particle in mm^3^.

### 2.4. Laboratory-Measured Shear Strength Properties

**Figure 7:**
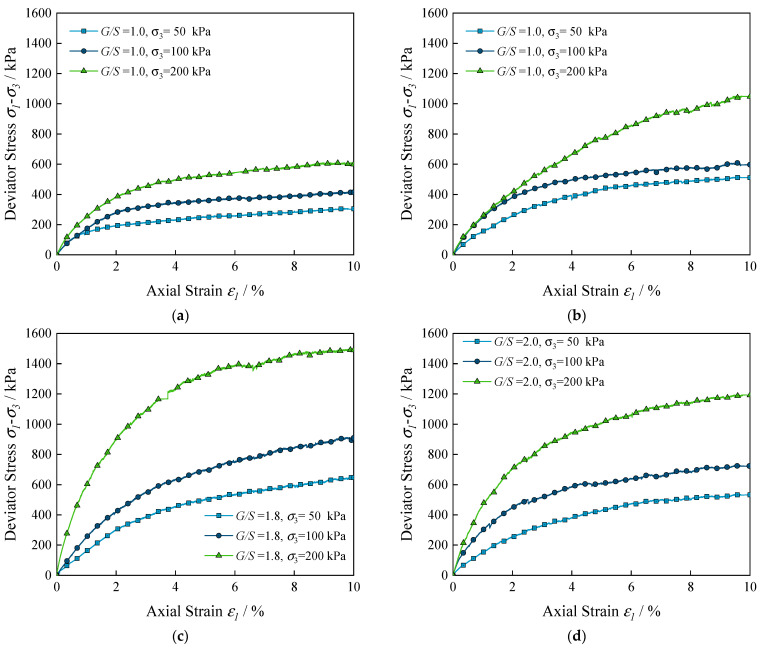

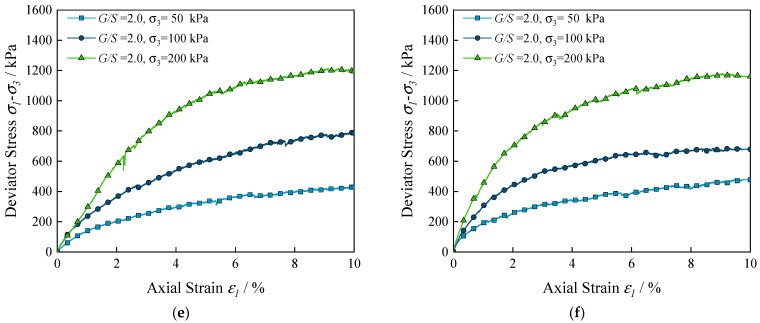
Laboratory-tested deviator stress versus axial strain curves of UPAB specimens with varying G/S levels: (**a**) G/S = 1.0; (**b**) G/S = 1.6; (**c**) G/S = 1.8; (**d**) G/S = 2.0; (**e**) G/S = 2.2; and (**f**) G/S = 2.5.

Since the stress–strain curves of UPAB specimens with different G/S values at different levels of confining pressure did not show obvious peaks, the axial strain of 10% was adopted as the criterion for judging the occurrence of shear failure of the specimens, and the peak deviatoric stress upon the occurrence of shear failure of the specimens at different levels of G/S values was determined accordingly. Figure 7 shows the stress–strain curves corresponding to six different G/S values of the specimens. It can be seen from Figure 7 that the deviatoric stress increases with increasing axial strain, and that the rate and magnitude of the increase of the deviatoric stress increase with increasing confining pressure.

**Figure 8:**
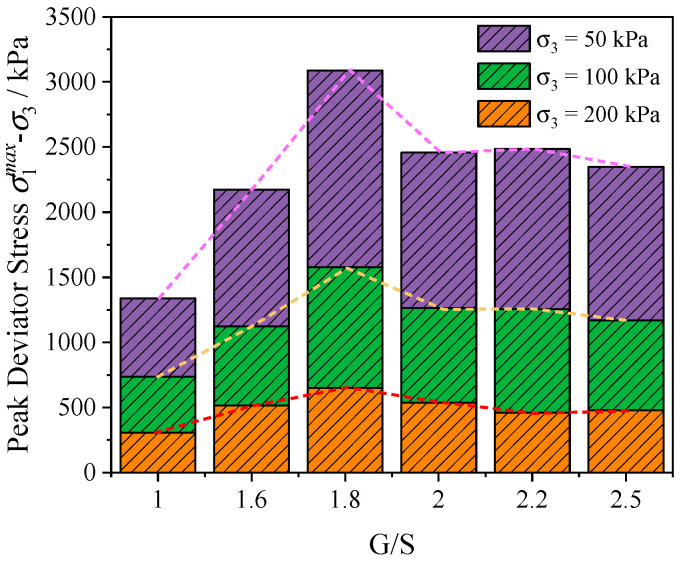
Histograms showing the values of peak deviator stress at failure obtained at different G/S levels from laboratory monotonic triaxial compression tests.

Figure 8 shows the values of peak deviatoric stress at failure for specimens with G/S values of 1.0, 1.6, 1.8, 2.0, 2.2, and 2.5 at different levels of confining pressure (50 kPa, 100 kPa, and 200 kPa). Apparently, great differences in peak deviatoric stress at failure exist among UPAB specimens with different G/S values even at the same level of confining pressure, and such differences become more obvious with increasing confining pressure. In addition, under different confining pressure levels of 50 kPa, 100 kPa, and 200 kPa, the maximum values of peak deviatoric stress at failure of different UPAB specimens all corresponded to the G/S value of 1.8, while the minimum values of peak deviatoric stress at failure all corresponded to the G/S value of 1.0. In other words, the UPAB specimen with G/S value of 1.8 has the best gradation (or particle packing structure), thus resulting in the highest shear strength; in contrast, the UPAB specimen with G/S value of 1.0 has relatively the weakest gradation or particle packing structure. Therefore, overall speaking, gradation is among the critical factors governing the shear strength properties of UPAB materials. Under the same level of confining pressure, the peak deviator stress at failure of UPAB materials increases first and then decreases with G/S value increasing from 1.0 to 2.5. This further confirms that the G/S parameter can be used to control the shear strength properties of UPAB materials and its value needs to be properly limited so that the relative proportions of coarse and find fractions can be reasonably balanced.

## 3. Description of the DEM Model Developed

### 3.1. General Principle of the DEM Model

The discrete element method (DEM) has been widely used due to its unique advantages in simulating the macroscopic and micromechanical behavior of granular materials [4,6,19,25,32,33,34,35,36,37,38,39]. However, due to the wide range of particle size distribution of UPAB materials, it is often computationally expensive to perform such DEM simulations, thus justifying the necessity to simplify the DEM model, especially the sizes of discrete particles simulated. Therefore, in this study, all fine particles smaller than 2.36 mm were replaced by 2.36 mm particles with the total mass conserved, which can greatly reduce computational time without significantly compromising computational accuracy. Qian et al. (2013) [39] studied the effect of shearing rate on shear strength from laboratory monotonic triaxial compression tests and found that shearing rate had an insignificant effect on shear strength test results. Therefore, the incremental displacement loading method is widely used in DEM simulations of laboratory monotonic triaxial compression tests. This method was also adopted for loading simulations in this study. The strength results from laboratory monotonic loading tests are not sensitive to the shearing rate; on the other hand, the use of greater shearing rate in the DEM simulations was found to not only improve the computational efficiency significantly, but also have trivial impact on the simulation results [40]. In order to significantly reduce computational time without compromising computational accuracy, the use of different shearing rates in laboratory tests (i.e., 1 mm/minute) and DEM simulations (i.e., 100 mm/second) was justified and adopted in this study.

### 3.2. Modeling Irregular Particle Shape

Belheine et al. (2009) [38] and Widuliński et al. (2009) [41] adopted spherical elements and introduced the rolling stiffness model to consider the influence of the actual shape of particles on the simulation results; however, there was a lack of consideration of the fabric anisotropy caused by the real particle shape. Hence, to study the micromechanical behavior of irregular particle assembly, the methods of particle size simplification, incremental displacement loading, and realistic particle shape characterization and simulation were adopted in this study to carry out DEM simulations with computational accuracy and time cost reasonably balanced.

**Figure 9:**
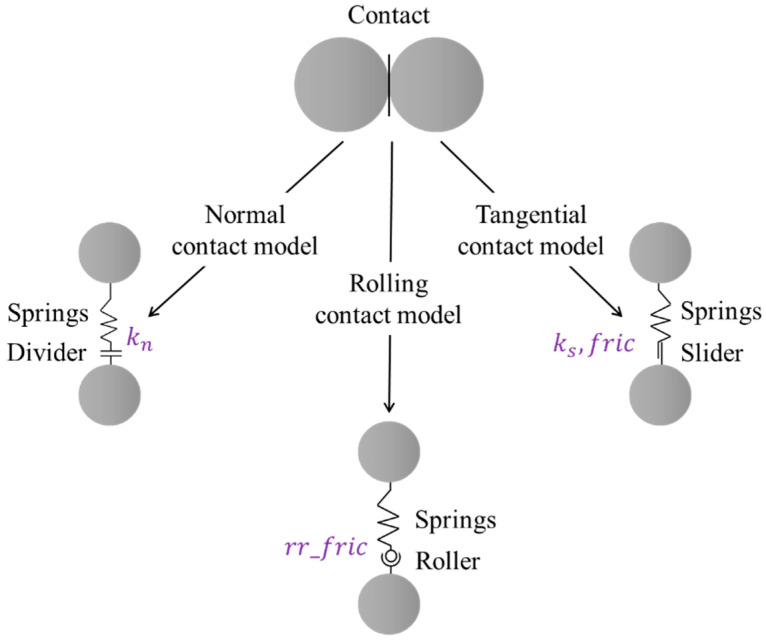
Illustration of micromechanical components of the rolling stiffness contact model used for DEM simulations.

According to statistical results of particle shape indices, many coarse particles selected for shape characterization were regarded convex in morphology; hence, the convex polyhedral elements were used in the DEM model to accommodate the computational efficiency. Note that the convex polyhedral elements can restore the irregular shape of real particles to a certain extent, but are relatively weak in representing their surface roughness. To address the lack of surface roughness representation of convex polyhedral elements, the rolling stiffness model shown in Figure 9 was used to describe the inter-particle contact behavior. The rolling stiffness model is similar to the linear stiffness model except for the fact that a rolling moment is added at the contact position to counteract the rolling effect. The moment applied increases linearly with the accumulation of relative rotation at the contact point. When the accumulated rotational moment exceeds its limit, the particles in contact start to rotate. As for the rolling stiffness model, a good rolling resistance mechanism can be achieved at the contact points without adding additional damping parameters. This can compensate for the deficiency of surface roughness representation by the convex polyhedrons used in the DEM model. The processes of generating convex polyhedral particles for the DEM model are illustrated previously in Figure 4.

The rolling stiffness contact model used the force-displacement law to calculate the forces and moments.

(8) $$F=F^{l}+F^{d}+F^{c},M_{c}=M^{r}$$

where $F^{l}$ is the normal linear elastic force, $F^{d}$ is the damping force, $F^{c}$ is the tangential friction, and $M^{r}$ is the rolling resistance moment. The normal linear elastic force, damping force and tangential friction force were updated according to the force-displacement law of the current stiffness model [42], and the rolling resistance moment was updated according to the following rules.

First, the increment of the rolling resistance moment was updated as follows.

(9) $$M^{r}=M^{r}-k_{r}\Delta \theta_{b}$$

where $\Delta \theta_{b}$ is the relative rotation increment and $k_{r}$ is the rotational contact stiffness.

(10) $$k_{r}=k_{s}R^{2}$$

where $k_{s}$ is tangential contact stiffness, and R is expressed as follows.

(11) $$\frac{1}{R}=\frac{1}{R_{1}}+\frac{1}{R_{2}}$$

where $R_{1}$ and $R_{2}$ are the radius values of particles No. 1 and No. 2 that are in contact, respectively.

When the stress states of particles changed, the motion parameters of particles were updated according to Newton’s second law of motion to simulate the physical phenomena of the particle assembly with rolling resistance.

**Figure 10:**
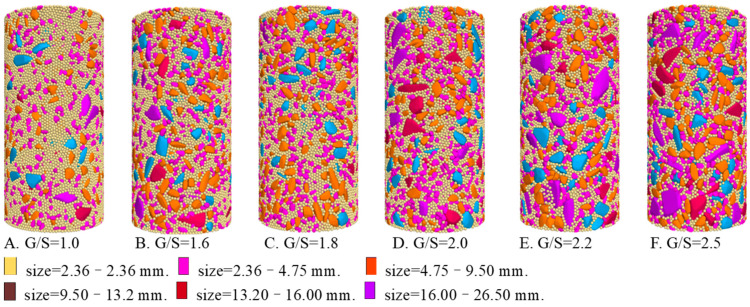
The compacted DEM specimens with different G/S levels.

After generating discrete DEM particles and selecting a reasonable inter-particle contact model, the DEM specimens generated were then compacted in consistency with laboratory tests. Figure 10 shows the compacted DEM specimens with different G/S values. It is worth mentioning that in order to enhance computational efficiency, the aggregate particles with sizes smaller than 2.36 mm were all replaced by those with sizes of 2.36 mm by following the principle of mass conservation. The dimensions of the DEM specimens were consistent with those of aforementioned laboratory ones. To enhance computational efficiency, particle sizes smaller than 2.36 mm were all simplified as 2.36 mm with the principle of equal mass. The porosity values of the DEM specimens with different G/S values were averaged from those of individual measurement circles at different locations, which were 0.220, 0.228, 0.235, 0.240, 0.247 and 0.257 for G/S values of 1.0, 1.6, 1.8, 2.0, 2.2 and 2.5, respectively. Such porosity results of the DEM specimens clearly satisfy the permeability requirement of drainable materials. To properly account for the effect of flexible rubber membranes used in laboratory tests, the coupling of discrete element method and finite difference method (DEM-FDM) was adopted, and the shell elements were used in the FDM model to simulate flexible rubber membranes. The effectiveness of this method was reported in the literature [43,44]. In this study, the incremental displacement loading method was adopted for the DEM model with the loading rate set to 100 mm/s, and the DEM specimens were subjected to confining pressure of 100 kPa to be consistent with laboratory tests.

### 3.3. Calibration of Micromechanical Parameters of the DEM Model

In DEM simulations, the calibration of micromechanical parameters should be first undertaken to ensure that the DEM model established could simulate physical laws reasonably well [41]. However, there is no definite or explicit mathematical relationship existing between macroscopic performance indicators and micromechanical DEM parameters. Therefore, it becomes necessary in practice to first calibrate such micromechanical parameters by adjusting their different combinations on a trial-and-error basis until the reasonably good match is achieved between actual laboratory testing and DEM simulation results. For brevity, the details of the calibration process were skipped and can be found elsewhere [45,46,47].

**Figure 11:**
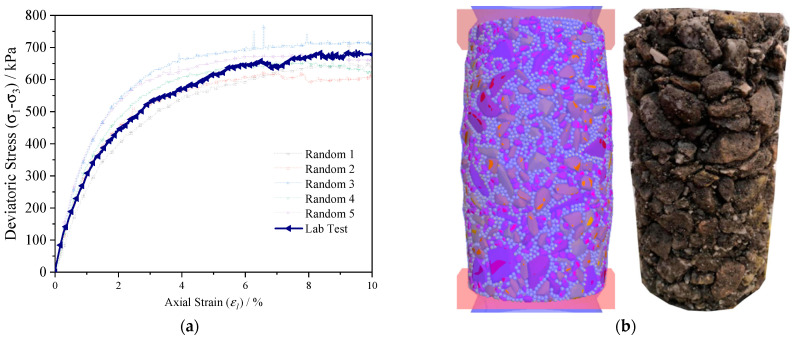
Illustration of (**a**) calibration results of the DEM model and (**b**) corresponding DEM and laboratory specimens at failure.

The laboratory specimen with G/S value of 2.5 was selected to calibrate micromechanical parameters of the DEM model. Since the realistic initial particle-packing structure (or initial fabric) of the real specimen was quite challenging if not impossible to capture and remained unknown in this study, it then became indispensable for the DEM simulations to numerically capture it as much as possible. For this purpose, five different random numbers were applied to generate varying initial particle-packing structures of virtual specimens, but the same set of micromechanical input parameters calibrated by laboratory testing results were adopted. Figure 11 shows the axial stress–axial strain curves from those five different DEM simulations conducted, where the random number yielding the best match between DEM-simulated and laboratory-measured results was eventually used in subsequent formal DEM simulations. Also shown in Figure 11 were the corresponding DEM and laboratory specimens upon shearing failure. It can be seen from Figure 11 that the axial stress–axial strain curve obtained from the laboratory test is enclosed by those from DEM simulations with different initial fabric scenarios, i.e., the realistic initial particle-packing structure of the real specimen can be numerically reproduced by using proper random numbers. The final calibrated micromechanical parameters are listed in Table 4, which can reflect macroscopic axial stress–axial strain behavior of UPAB materials.

## 4. Results and Discussion

### 4.1. Analysis of Deviatoric Stress–Axial Strain Characteristics

**Figure 12:**
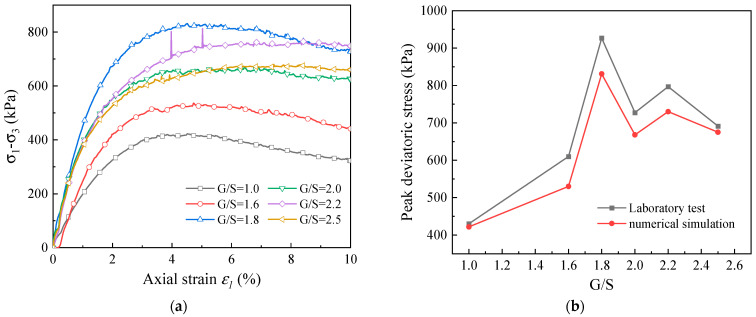
DEM-simulated results of monotonic triaxial compression tests: (**a**) deviator stress versus axial strain curves for different G/S levels and (**b**) comparison of lab-measured and model-simulated peak deviator stress for different G/S values.

Figure 12a shows the deviatoric stress–axial strain curves of UPAB materials with different G/S values as predicted by the DEM model established. It can be seen that the deviatoric stress first increases and then decreases with increasing axial strain for different G/S levels. The specimen with the smallest G/S value of 1.0 (i.e., the greatest fines content) exhibits the lowest shear strength, while the peak deviator stress of the specimen with G/S value of 2.5 (i.e., the least fines content) is not the largest. This indicates that the influence of fines content on shear strength of UPAB materials is not linear. Figure 12b plots both laboratory-measured and model-predicted curves of peak deviator stress against G/S value, from which a reasonably accepted match is observed. It also shows that the specimen with G/S value of 1.8 exhibits the maximum peak deviatoric stress both experimentally and numerically, whereas the specimen with G/S value of 1.0 exhibits the minimum peak deviatoric stress.

**Figure 13:**
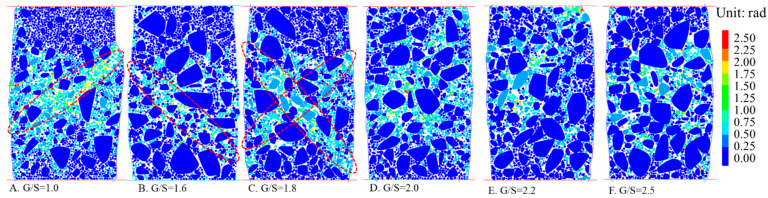
Sectional contour plots showing internal particle rotation for specimens with different G/S values upon shear failure.

Figure 13 shows contour plots of internal particle rotation within identical representative sections of different G/S value DEM specimens upon shear failure. It can be seen that when the axial strain reaches 10%, the formation of the shearing band is related to the rotation of particles, and that the shearing band becomes less obvious for greater G/S values. In contrast, the smaller the G/S value is, the more obvious the rotation of particles is, and the shearing band is formed in the area where larger rotation occurs. This indicates that the rotation of particles is the main reason for the instability of specimens with greater fines content. When the content of fine particles is low, the rotation of particles is clearly suppressed, and the relative sliding effect among particles is enhanced. The rotation effect of particles is found related to particle size, i.e., the smaller the particle size is, the higher the rotation tendency is. Further, proper reduction of the content of fine particles can strengthen the sliding effect among particles, in other words, as the G/S value increases, the mode of particle movement within the shearing band of the DEM specimen transitions from rotation to relative sliding, and the failure mode also changes from shearing failure to relative sliding failure of local interfaces.

### 4.2. Coordination Number and Deviator Fabric

The evolution process and mechanism of microscopic inter-particle contact characteristics of UPAB specimens with different G/S values can be quantitatively analyzed and revealed from DEM simulation results of coordination number and deviatoric fabric. Specifically, the coordination number reflects the average number of particles in contact with each of all the particles in the aggregate assembly, which can be calculated according to Equation (12).

(12) $$P=\frac{\sum_{1}^{n}n_{i}}{N_{num}}$$

where $n_{i}$ is the number of contact points between each particle and its surrounding particles, and $N_{num}$ is the total number of particles in the aggregate assembly.

**Figure 14:**
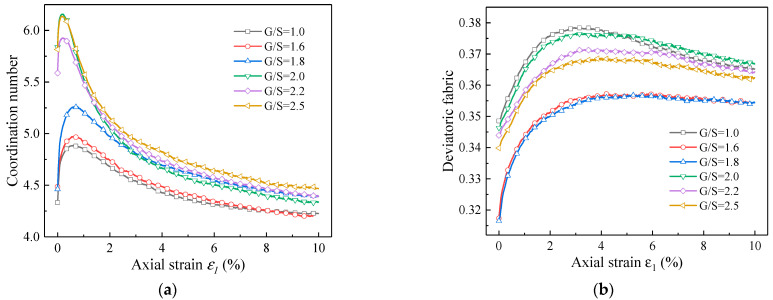
Model-predicted curves of (**a**) coordination number versus axial strain and (**b**) deviatoric fabric versus axial strain.

Figure 14a shows the DEM model predicted curves of coordination number against axial strain for UPAB specimens with different G/S values. As the axial strain increases, the coordination number of DEM specimens with different G/S values first increases and then decreases to a stable value. This is actually similar to the results reported by [23]. In the stage of increasing coordination number, the specimens were continuously compacted, and the number of contact points among particles and the degree of compaction increase. During this stage, the axial strain is dominant. After the coordination number reached the peak, the contact force chain among particles began to fail, leading to the decrease in the coordination number. It can be found from the comparison between Figure 14a and Figure 12a that the axial strain value corresponding to the peak coordination number is smaller than that corresponding to the peak deviatoric stress at failure. The failure of internal contact force chains of the specimens implies that the internal plastic deformation starts to develop; in other words, the failure of contact force chains is the microscopic manifestation of the development of the plastic zone within the specimens. Overall, the initial coordination number of DEM specimens with different G/S values is greater than 4, indicating that the initial degree of compaction of the specimens is acceptable. The coordination number of the specimen with G/S = 1.0 is the lowest, indicating that the greatest number of “floating” particles exist within the specimen and no stable particle skeleton is formed. This leads to the lowest shear strength of the specimen with G/S = 1.0. On the other hand, the coordination number of the specimen with G/S = 2.0 is the highest, but its shear strength is not the largest. The reason is that the content of fine particles in the specimen with G/S = 2.0 decreases and the content of coarse particles increases. Although the average contact points of particles within the specimen increase to a certain extent, the optimal load-carrying aggregate skeleton is not formed yet within the specimen, which can be confirmed by the results in Figure 14b.

The mechanical responses of UPAB materials are macroscopic manifestation of the interaction and evolution of all contact points of internal particles. By calculating the deviatoric fabric, the degree of anisotropy of internal inter-particle contact of the specimens can be quantified, and the evolution law of anisotropic characteristics of the specimens with different G/S values can be disclosed during the entire process of monotonic triaxial compression test. The definition of deviatoric fabric is formulated as follows [48,49,50,51,52].

(13) $$\varphi_{d}=\sqrt{{\left(\varphi_{1}-\varphi_{2}\right)}^{2}+{\left(\varphi_{1}-\varphi_{3}\right)}^{2}+{\left(\varphi_{2}-\varphi_{3}\right)}^{2}}$$

where $\varphi_{1}$, $\varphi_{2}$ and $\varphi_{3}$ represent the major, intermediate, and minor principal values of the contact force in each of the three orthogonal contact directions.

Figure 14b shows DEM model predicted curves of deviatoric fabric versus axial strain for specimens with different G/S values during monotonic triaxial compression tests. It can be seen that the deviatoric fabric first increases and then decreases to a stable value with increasing axial strain, and that the contact points of particles tend to develop along the vertical direction to sustain greater deviatoric stress. After shearing failure, the rearrangement of contact points of particles within the specimen weakens in the vertical direction, resulting in extensional deformation. Among the DEM specimens, the one with G/S = 1.8 has the lowest deviatoric fabric, thus indicating that the distribution of the magnitudes of the contact forces on three orthogonal directions is more uniform and the degree of anisotropy is the lowest. That is, the particle packing structure of the specimen with G/S = 1.8 is more stable than those of other specimens, which is also the main reason for the maximum peak deviator stress at failure achieved for G/S = 1.8. In contrast, the specimen with G/S = 1.0 has the largest deviatoric fabric, i.e., the contact forces inside the specimen are the most non-uniform and the degree of anisotropy is the highest, which leads to the lowest peak deviator stress at failure. Therefore, it can be inferred that the influence of the content of fine particles on shear strength of UPAB materials is manifested by the change in particle packing structure.

### 4.3. Evolution Characteristics of Particle Contact Fabric

As an important index to characterize the anisotropy, fabric anisotropy has a great influence on the strength and deformation behavior of unbound granular materials [53,54]. The anisotropy of soils can be divided into two different types, i.e., the primary anisotropy and stress-induced anisotropy. Primary anisotropy describes the anisotropy caused by particle alignment during geotechnical deposition, while stress-induced anisotropy visualizes the anisotropy exhibited by the geotechnical body under external loading. In other words, the internal structural and physical properties of materials subjected to external loading evolve with time, which is an important index to characterize the strength and deformation behavior of materials under external loading. In this section, the initial anisotropy of DEM specimens with different G/S values in the stage of zero axial strain was studied, and three-dimensional rose diagrams in the spherical coordinate system were subsequently drawn to quantitatively characterize both primary and stress-induced anisotropy characteristics of specimens with different G/S values and to reveal the influence of the content of fine particles on anisotropy characteristics during the shearing process.

**Figure 15:**
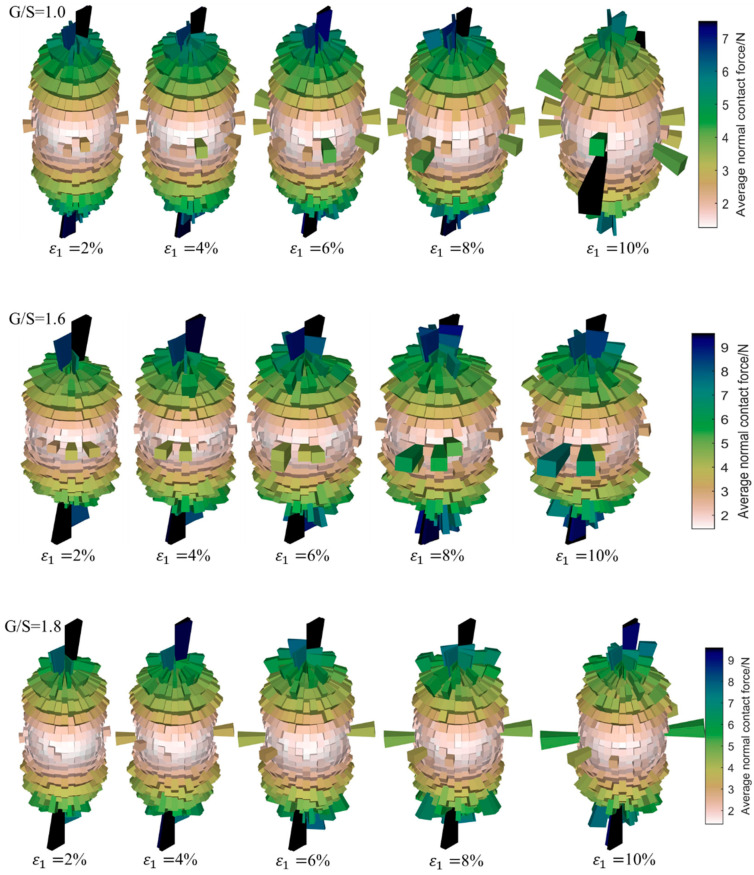

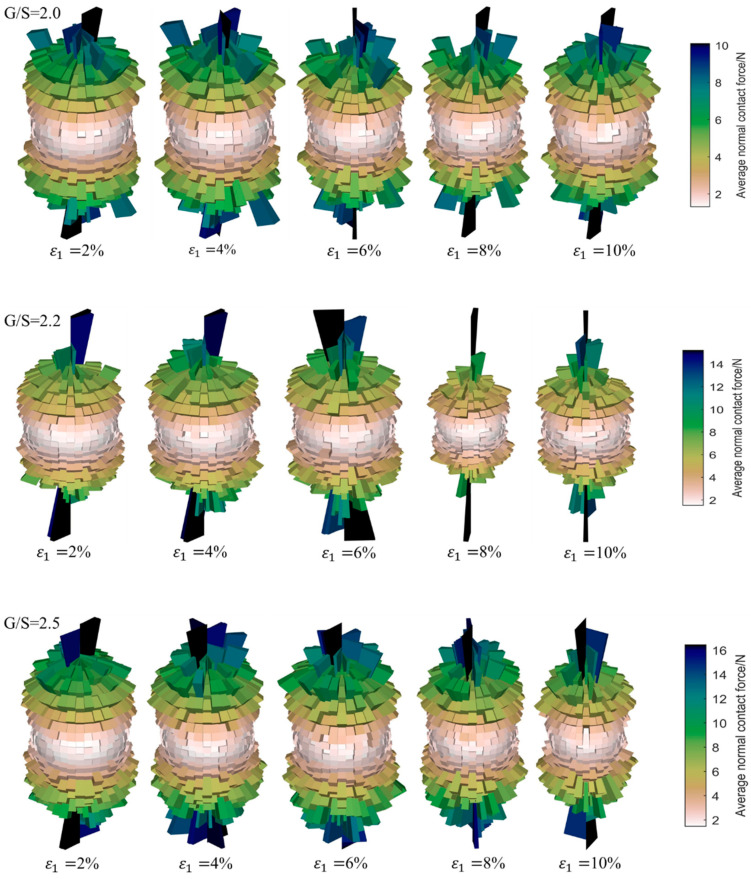
Three-dimensional rose diagrams showing the evolution of normal contact forces during the shearing process for specimens with different G/S values.

Figure 15 shows the magnitudes and orientations of average normal contact forces for specimens with different G/S values at different levels of axial strain, which characterizes the development evolution of the stress-induced anisotropy of each DEM specimen under 100-kPa confining pressure. It can be seen that the anisotropy of average normal contact forces at the initial stage of shear loading is predominant in the vertical direction, i.e., the principal contact forces among particles are mainly concentrated in the vertical direction. With the progress of shear loading, the specimens began to deform laterally; in addition, lateral contact forces increase gradually, which shows the dilatancy of the specimens. As the shearing process further continues, the degree of anisotropy of the specimens with different G/S values increases gradually, and the rose diagrams of average normal contact forces transition gradually from being nearly spherical to elongated along the axial direction. This indicates that the shear loading promotes the development of fabric anisotropy in the specimens. The major principal contact forces in the transverse directions decrease with increasing G/S value, and the majority of the shear load is sustained by vertical inter-particle contact forces. However, there seems to exist a thresholding G/S value of 1.8, and the development of transverse inter-particle contact forces becomes less obvious for G/S values exceeding beyond this threshold of 1.8.

**Figure 16:**
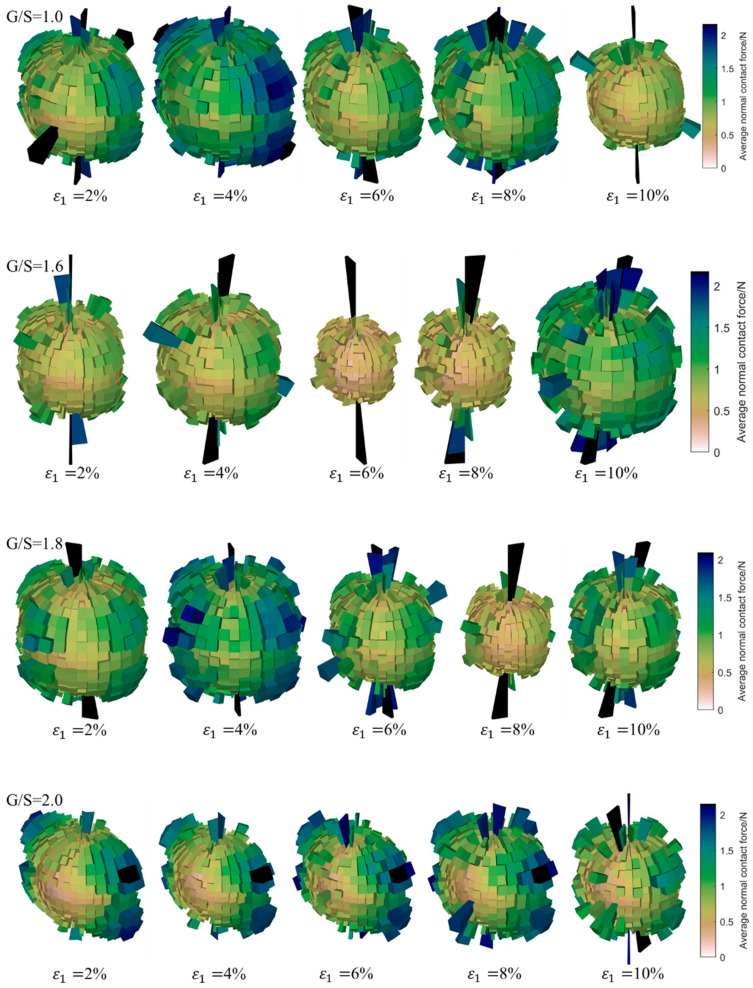

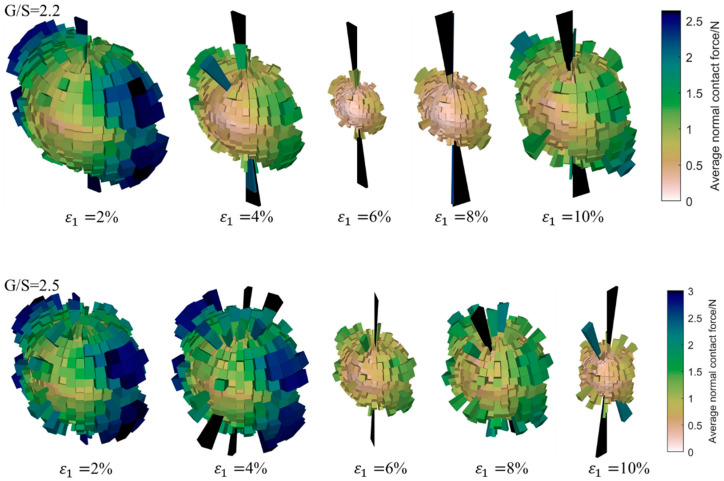
Three-dimensional rose diagrams showing the evolution of tangential contact forces during the shearing process for specimens with different G/S values.

Figure 16 shows the evolution of tangential inter-particle contact forces of the specimens with different G/S values during the shearing process under the application of 100-kPa confining pressure. It can be seen that when the axial strain is small, the specimens with more fine particles show less fabric anisotropy of inter-particle contact forces, while the specimens with less fine particles show obvious deformation in the direction of principal normal contact forces. As the shear loading further continues, the tangential fabric of specimens with different G/S values shows obvious anisotropy, and the direction of maximum tangential contact force deflects considerably. When the content of fine particles is large, only part of the tangential contact forces is obvious, which is related to the dominant relative rotation of fine particles. As the content of fine particles decreases, the fabric of tangential contact forces exhibits a relatively apparent dominant direction, which is related to increasing relative sliding among particles. When G/S value is greater than 1.8, the fabric of tangential contact forces shows the formation of multiple dominant directions with increasing axial strain, thus indicating that the shearing band within the specimens is not concentrated or unique. This may be attributable to the fact that no obvious shearing band was observed from macroscopic failure.

Overall speaking, it can be found that the anisotropy evolution law of inter-particle contact forces during the shearing process is similar to that of deviatoric fabric. The normal contact forces first increase and then decrease with increasing axial strain, indicating that the evolution law of normal contact forces and deviatoric fabric is the microscopic reflection of macroscopic shear strength behavior. Before the deviatoric stress–axial strain curves reach their respective peak values, the re-arrangement of particles occurs within the specimens so that the dominant load-carrying aggregate skeleton tends to orient towards the direction of deviatoric stress. Through this rearrangement and self-assembly process, the energy associated with external loading is dissipated. This change is approximately linear from mechanical perspective; hence, it corresponds to the elastic stage on the macroscopic deviatoric stress–axial strain curve, and the applied external energy is partly converted into elastic strain energy stored. In the specimens with more fine particles, the rolling action of fine particles plays a dominant role in the subsequent energy consumption during the instability stage, and the macroscopic responses of the specimens feature increasing plastic strain and the formation of a clear shearing band. The results show that the greater the content of fine particles is, the more pronounced the transverse inter-particle contact forces and the dilatancy upon yielding are. For specimens with a lesser proportion of fine particles, the relative sliding effect among particles and the rolling effect of fine particles play a leading role in the subsequent energy consumption, but multiple dominant directions of principal tangential contact forces are formed, thus leading to the lack of apparent shearing band within the specimens. When the axial strain is low, the fabric anisotropy of tangential inter-particle contact forces exhibits increasingly obvious directionality with decreasing content of fine particles, which is related to increasing proportion of relative sliding among particles. Those particles are constrained by tangential friction at the initial stage, while the friction increases and the sliding effect decreases with increasing axial stress. At the high level of axial strain, the directionality of principal tangential contact forces becomes insignificant, which leads to disordered fabric orientation. This indicates that localized sliding among particles gradually becomes dominant, i.e., plastic deformation occurs.

During the shearing process, the normal contact force chains among particles are further strengthened. The lower the content of fine particles is, the more profound the strengthening effect is. This is related to the reduction in the content of fine particles and the load-sustaining aggregate skeleton formed within the specimens. When the specimens start to experience plastic yielding gradually, the fabric of normal inter-particle contact forces is anisotropic, the strengthening effect in the main direction becomes weakened, but transverse contact force chains become strengthened. This indicates that the specimens start to undergo macroscopic dilatancy with particles gradually losing contacts during the deformation process. In other words, the loss of the inter-particle contact force chains serves the early sign of plastic zone development within the specimens. The plastic yielding of specimens at the high level of axial strain could be related to the change of topological packing structure of the particle assembly, which is manifested as the rotation of particles inside the specimens with more fine particles and as the relative sliding among particles inside the specimens with less fine particles. The changes of both topological packing structures could lead to irreversible plastic deformation of the specimens.

### 4.4. Rotational Characteristics of Particles

**Figure 17:**
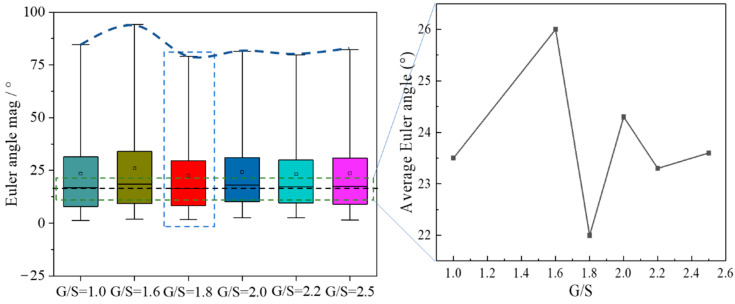
The variation of average Euler angles of particles with G/S level.

**Figure 18:**
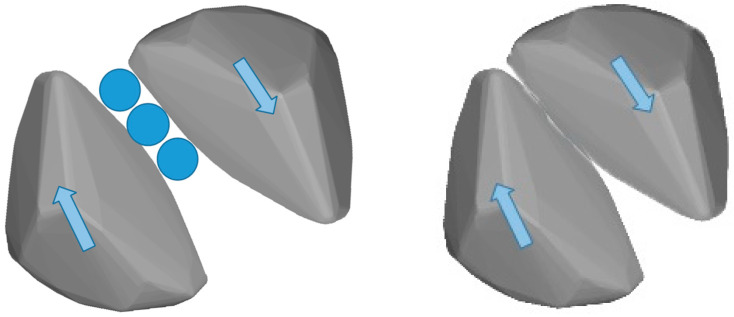
Illustration of rolling and relative sliding interactions between particles causing loss of contact.

Figure 17 shows the average Euler angles of the particles in the DEM specimens with different G/S values upon shearing failure. It can be seen that the average Euler angle of particles in the specimen with G/S = 1.8 is the smallest, which implies that the spatial arrangement and packing structure of particles inside this specimen are the most stable during the shearing process. Since the content of fine particles and inter-particle contacts are relatively optimal, the relatively weak rotation of particles is observed. Nevertheless, the specimen with G/S = 1.8 still exhibits relatively clear, macroscopic shearing band due to the existence of a certain proportion of fine particles. The average Euler angle of particles in the specimen with G/S = 2.0 is greater than that in the specimen with G/S = 1.8, for which the main reason may be that the reduced content of fine particles of the former specimen leads to more “floating” fine particles in the relatively larger air voids, and that the floating particles rotate considerably under the application of deviatoric stress. Therefore, it can be inferred that the G/S value of 1.8 can be used as the threshold distinguishing skeleton dense packing structure from skeleton gap packing structure. For clarity, the rolling and relative sliding interactions between particles are illustrated in Figure 18 that could cause loss of interparticle contact.

**Figure 19:**
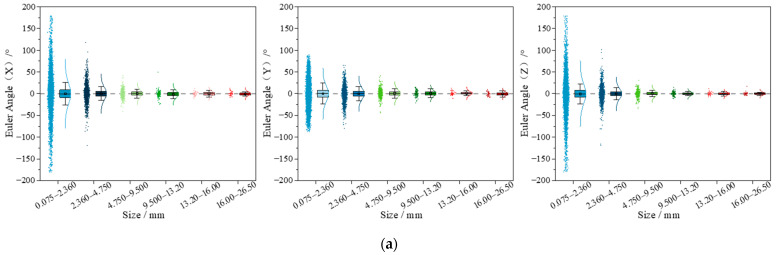

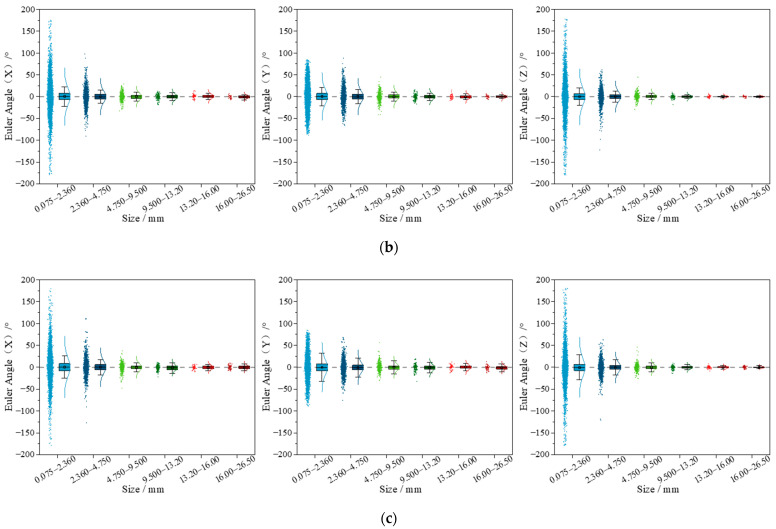
DEM-simulated Euler angles of particles within different size ranges for UPAB specimens with (**a**) G/S = 1.0, (**b**) G/S = 1.8, and (**c**) G/S = 2.5.

In addition, the Euler angles of particles with different sizes were calculated to quantitatively describe their rotational movement characteristics. The Euler angle is a rotational motion parameter defined based on the local coordinate system of particles. In this study, the Euler angles in the X, Y, and Z directions of particles within different size ranges were calculated and analyzed for UPAB specimens with G/S = 1.0, G/S = 1.8, and G/S = 2.5, respectively. It can be seen from Figure 19 that the UPAB specimens with different G/S levels generally exhibit similar rotational motion characteristics in all three different directions, i.e., the greater the particle size is, the weaker the particle rotation motion is, and vice versa. For particles within each of the two fine size ranges (i.e., 0.075–2.36 mm and 2.36–4.75 mm), their Eulerian angles in X, Y, and Z directions are observed to be much more widely distributed with much greater magnitudes, respectively, which indicates that the rotation of particles within such two size ranges is much more pronounced and intense. On the other hand, for particles within each of the coarse size ranges (i.e., larger than 9.5 mm), their Eulerian angles in X, Y, and Z directions are observed to be much more insignificant and narrowly distributed with much smaller magnitudes, respectively, i.e., the rotation motion of particles within such size ranges is dramatically weakened. Therefore, the size range of 4.75–9.5 mm can be regarded as the thresholding boundary separating rolling- and sliding-dominating particle motion patterns. Therefore, the contents of particles ranging from 4.75–9.5 mm and smaller than 4.75 mm need to be well controlled, respectively.

## 5. Summary and Conclusions

In this paper, both laboratory monotonic triaxial compression tests and accompanying discrete element method (DEM) simulations were carried out for unbound permeable aggregate base (UPAB) materials, with their gradations designed by the gravel-to-sand (G/S) concept proposed from particle packing theory. The microscale fabric evolution and instability mechanism of macroscopic shear strength behavior of UPAB materials in triaxial compression were studied and disclosed. The main conclusions are summarized as follows.

(1) The gradation control parameter G/S has a pronounced control effect on the shear strength of UPAB materials. As the G/S value increases, the shear strength of UPAB specimens first increases and then decreases, and the shear strength of the specimen with G/S = 1.8 is the greatest. The influence of fine fraction on the shear strength is not linear. The movement characteristics of fine particles directly affect macroscopic shear strength of UPAB specimens, i.e., the increase of the sliding ratio among particles has a positive effect on improving the shear strength.
(2) The UPAB specimen with G/S = 1.8 exhibits the lowest meso-scale anisotropy, the best particle packing structure, and thus the highest macroscopic shear strength, while the UPAB specimen with G/S = 1.0 shows opposite trends. From the results of coordination number and fabric evolution, the plastic yielding of UPAB specimens are manifested by the failure of interparticle force chains at the early stage and are then related to the changes in topological structure of particle packing at the later stage (i.e., the rolling and sliding movement patterns of particles).
(3) The average Euler angles of particles in the UPAB specimen with G/S = 1.8 are the smallest, indicating that the interlocking among particles is better and the rolling ratio among particles decreases due to its optimal particle packing structure. The specimen with G/S = 2.0 has fewer fine particles than that with G/S = 1.8, but the average Euler angles of the former are greater than those of the latter, thus indicating that the particle packing and arrangement structure affects particle motion during the shearing process.
(4) G/S = 1.8 can be used as the threshold boundary separating skeleton dense and skeleton gap type packing structures, whereas the particle size range of 4.75–9.5 mm can be regarded as the threshold boundary separating rolling- and sliding-dominating particle motion. Properly reducing the content of particles smaller than 4.75–9.5 mm could help increase the sliding ratio among particles and thus improve the shear strength stability. The rationality of the G/S concept is verified from the perspective of meso-scale particle movement and fabric evolution, of which both govern macroscopic stability in triaxial compression.

## Figures and Tables

**Table 1:** Gradation design scheme and corresponding gradation parameters.

G/S	*D*_max_/mm	Porosity (*n*)	Coefficient of Uniformity (*C_u_*)	Coefficient of Curvature (*C_c_*)
1.0	26.5	0.32	270.22	3.55
1.6	26.5	0.51	29.09	1.97
1.8	26.5	0.56	23.64	2.08
2.0	26.5	0.61	18.09	1.90
2.2	26.5	0.65	14.46	1.74
2.5	26.5	0.71	11.63	1.63

**Table 2:** The optimal moisture content (OMC) and maximum dry density (MDD) results for the five different gradations representing different levels of particle breakage.

G/S	Maximum Dry Density MDD (g/cm^3^)	Optimum Moisture Content OMC (%)
1.0	2.338	4.710
1.6	2.340	4.760
1.8	2.351	4.762
2.0	2.271	4.400
2.2	2.269	4.660
2.5	2.270	4.850

**Table 3:** Calculation results of typical shape indices of selected particles.

Particle Size/mm	Shape Index					
	$C_{n}$	$C_{s}$	$C_{ns}$	$E_{x}$	$E_{xe}$	$R_{e}$
26.5–19.0 (19 pcs *)	0.780	0.721	0.746	0.889	0.863	0.838
19.0–16.0 (21 pcs)	0.711	0.790	0.744	0.883	0.867	0.837
16.0–13.2 (21 pcs)	0.692	0.783	0.730	0.874	0.875	0.836
13.2–9.50 (30 pcs)	0.719	0.708	0.707	0.877	0.870	0.835
9.50–4.75 (27 pcs)	0.667	0.770	0.709	0.871	0.880	0.837
4.75–2.36 (9 pcs)	0.657	0.744	0.696	0.881	0.826	0.809
Average	0.704	0.753	0.722	0.879	0.864	0.832
Median	0.724	0.765	0.726	0.881	0.875	0.840
Maximum	0.962	0.992	0.895	0.940	0.927	0.902
Minimum	0.352	0.448	0.530	0.730	0.581	0.565

Note: * denotes number of particles selected for each of the size ranges to characterize and quantify particle shape.

**Table 4:** The calibrated micromechanical parameters of the DEM model.

Symbol	Parameter Definition	Value (Particle–Particle/Particle–Wall)
*Kn*	Normal stiffness (N/m)	5 × 10^7^/1 × 10^8^
*Ks*	Tangential stiffness (N/m)	5× 10^7^/1 × 10^8^
*Rr_fric*	Rolling friction coefficient	0.1/0.0
*Fric*	Friction	0.35/0.1

## Data Availability

Not applicable.
